# The Physical Properties of Dental Amalgams

**Published:** 1898-12

**Authors:** T. B. Hitchcock

**Affiliations:** Boston.


ARTICLE VI.

### THE PHYSICAL PROPERTIES OF DENTAL AMALGAMS.

THE LATE PROF. T. B. HITCHCOCK, M. D., D. M. D., BOSTON.

[Extracts from a paper in the *British Journal of Dental Science*, Vol. XVIII., June issue, showing that excess of mercury and washing in preparing amalgam fillings is unscientific.]

"The most diverse results may be obtained by the manner in which the various amalgams are prepared; even the best may be rendered worthless by improper manipulation. In order to obtain the best results, the mercury used should be only sufficient to cause the mass to cohere A true amalgam should contain no free mercury, but as usually mixed there is a very great excess. If only a small proportion be used, the amalgam hardened very much sooner. If the cavity to be filled will permit it, the amalgam should be used very dry and even granular, and although it is worked with some difficulty, the result obtained is much more satisfactory. . . . .

"When an excess of mercury is used in amalgam it always comes to the surface, and an unequal plug is the result; moreover, the tendency to contract or assume the spheroidal shape is very largely increased. Mr. G. H. Mankins, lecturer on metallurgy in the London School of Dental Surgery, well states the case as follows :

" Amalgams, as a rule, are compounded of a metal and mercury, which compound is further dissolved in an excess of mercury; and although this excess under pressure is largely squeezed out, as in larger ore operations for gold or silver separating, yet it is very difficult to do this to such an extent that no free mercury is retained. And, indeed, by much the same action as when one squeezes a sponge containing a fluid, you get the mercury forced to the surface, and thus, in your operations, what is so forced between the sides of the cavity and the plug, by slow and gradual evaporating; is liable to leave a minute interspace.

"For when too much has once been added, any pressure to which we can subject it with chamois-skin and pliers will fail to remove all excess. The best results will only be obtained with a very much less amount than will remain after any attempt to remove the surplus by pressure. . . . . . . .

"If only the proper quantity of mercury is used in making the amalgam, there is but a very small amount of oxides present, and consequently less necessity exists for their removal. By an excess of mercury we get just what washing is designed to remove."

"Tests made by Mr. Fletcher with amalgam plugs containing an excess of mercury and with amalgam which had been washed gave the following results, viz,: The plugs mixed with an excess of mercury are distinctly more imperfect and have a decidedly greater shrinkage in every case. The proportion of mercury left in after thorough compression with powerful pliers is about double what is necessary to make a good amalgam, and the excess of mercury cannot be squeezed out without making the amalgam so hard as to be unworkable; also the mercury comes to the surface of the filling, making an unequal alloy. As regards washing, when once wet I have *never* in a single case been able to get the amalgam properly dry without an amount of trouble such as would not be taken by ninety-nine operators out of a hundred, and the water works to the surface in packing, Several plugs put in smooth parallel tubes with an amalgam which had been washed, slipped out after hardening on the slightest force being applied. With the same amalgams which had not been washed this was most decidedly never the case under any possible circumstances with amalgams of moderate shrinkage."

"My own experiments made under the same conditions agree with the foregoing results by Mr. Fletcher." "Although Mr. Fletcher is a manufacturer, his investigations and experiments have been so extensive that his testimony is too valuable not to be used."—*Western Dental Journal.*

